# Structural defects in a nanomesh of bulk MoS_2_ using an anodic aluminum oxide template for photoluminescence efficiency enhancement

**DOI:** 10.1038/s41598-018-25045-z

**Published:** 2018-04-27

**Authors:** TaeWan Kim, DongHwan Kim, Chan Ho Choi, DaeHwa Joung, JongHoo Park, Jae Cheol Shin, Sang-Woo Kang

**Affiliations:** 1 Advanced Instrumentation Institute, Korea Research Institute of Standards and Science, Daejeon, 34113 South Korea; 2 Department of Physics, Yeungnam University, Gyeongsan, 38541 South Korea; 3 National Institute for Nanomaterials Technology, Pohang, 37673 South Korea; 4 Department of Electrical Engineering, Kyungpook National University, Daegu, 41566 South Korea; 5 Department of Next-generation Device Engineering, University of Science and Technology, Daejeon, 34602 South Korea

## Abstract

Two-dimensional (2D) materials beyond graphene have attracted considerable interest because of the zero bandgap drawbacks of graphene. Transition metal dichalcogenides (TMDs), such as MoS_2_ and WSe_2_, are the potential candidates for next 2D materials because atomically thin layers of TMDs exhibit unique and versatile electrical and optical properties. Although bulk TMDs materials have an indirect bandgap, an indirect-to-direct bandgap transition is observed in monolayers of TMDs (MoS_2_, WSe_2_, and MoSe_2_). Optical properties of TMD films can be improved by the introduction of structural defects. For example, large-area spatial tuning of the optical transition of bulk MoS_2_ films is achieved by using an anodic aluminum oxide (AAO) template to induce structural defects such as edge- and terrace-terminated defects in a nanomesh structure. Strong photoluminescence emission peaks with a band gap of 1.81 eV are observed, possibly because of radiative transition at the defect sites. This work shows that the AAO template lithography method has potential for the production of homogenous large-scale nanomesh structures for practical semiconductor processing applications in future MoS_2_-based electronic and optical devices.

## Introduction

Atomically thin layers of semiconducting transition metal dichalcogenides (TMDs), new semiconducting beyond graphene, have attracted attention for next-generation optoelectronics and electronics owing to their distinctive optical and electrical characteristics, providing tremendous opportunities in the fabrication of nanoscale optical and electrical devices^1–5^. In particular, two-dimensional (2D) monolayer (ML) TMDs have significantly enhanced optical properties owing to their indirect-to-direct band gap transition^6–12^. Previous studies have reported the indirect-to-direct band gap transition in monolayers of TMDs MX_2_ (M = Mo, W and X = S, Se, Te) [i.e. MoS_2_ (1.3 to 1.89 eV)^10^, MoSe_2_ (1.41 to 1.55 eV)^11^, MoTe_2_ (0.88 to 1.02 eV)^9^, WS_2_, (1.43 to 1.97 eV)^12^, and WSe_2_ (1.4 to 1.65 eV)^12^] materials. The synthesis of large scale monolayers of TMDs using chemical vapor deposition (CVD), however, is difficult to achieve due to the problems in controlling the highly uniform atomic monolayer^12,13^. An alternative method for tuning the band gap of the 2D materials is engineering a nanostructural defect using a template patterned with nano-sized holes and a strain-induced using a textured structure and mechanical strain^14–17^. It is well established that structural defect engineering in graphene can significantly affect its optical and electrical properties^13,14,18–20^. Nanomesh graphene using an anodic aluminum oxide (AAO) template and block copolymer lithography exhibit band gap opening, which overcomes the material’s limitation of zero bandgap in graphene^13,14^. However, in TMD materials, structural defect engineering is not straightforward because of their complicated structure and alloy system. There are only few reports on the effect of structural defects on electrical and optical properties of monolayer MoS_2_^21,22^.

Here, a nanomesh-MoS_2_ structure was generated using an anodic aluminum oxide (AAO) template. The AAO template is used widely to provide large scale nano-patterned structures. The self-organization process by anodization can lead to a densely packed nano-sized hole array. The hole size and neck width of the AAO template was tuned from 20 nm to 250 nm, resulting from anodization in an oxalic acid solution. Note that bulk MoS_2_ employing a nano-hole array structure exhibited remarkable enhancement in luminescence efficiency, because of the presence of structural defects. More importantly, this film exhibited strong photoluminescence (PL) emission peaks with a band gap energy of 1.81 eV, possibly as a result of dominantly radiative recombination excitons at defect sites. This paper reports the potential of the AAO template lithography method for the production of a homogenous large-scale nanomesh structure for practical semiconductor processing applications in future MoS_2_-based electronic and optical devices.

## Results and Discussion

**Figure 1:**
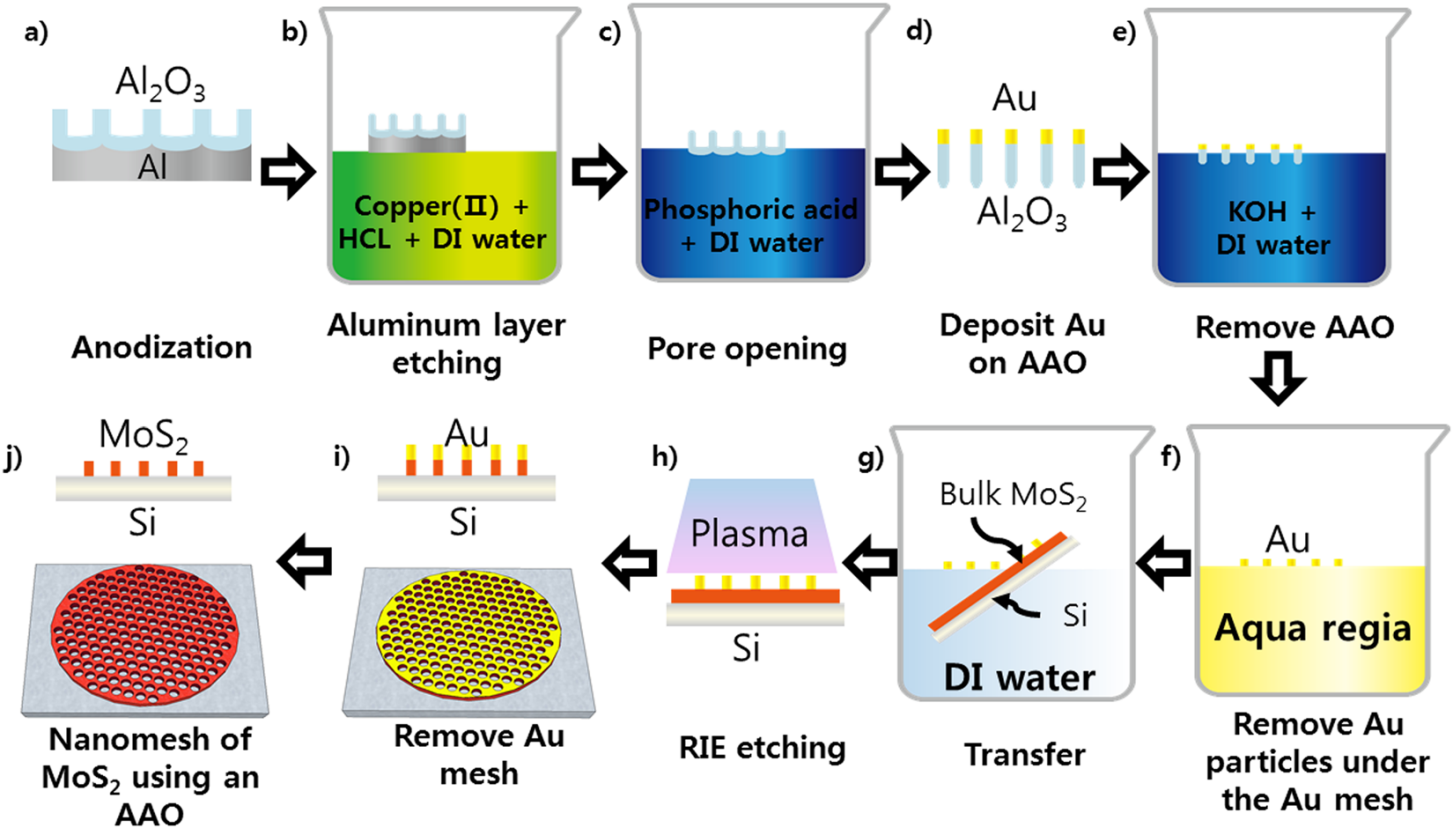
Schematic diagram of the fabrication of a MoS_2_ nanomesh.

Multilayer MoS_2_ flakes were obtained by the mechanical exfoliation of bulk MoS_2_ crystals and transferred to SiO_2_/Si substrates. Figure 1 shows the process sequence to form a MoS_2_ nanomesh. First, a nanoporous Al_2_O_3_ membrane was achieved by the anodization of a high purity aluminum disk (99.999%). Details for achieving the nanoporous Al_2_O_3_ membrane are provided in the experimental section. The residual aluminum layer was then etched selectively in a mixture solution of CuCl_2_ (13 g), HCl (200 mL), and deionization (DI) water (400 mL). In general, the nanopores on top of the Al_2_O_3_ layer were rough because of the massive cracks resulting from the continuous growth of the strained Al_2_O_3_ layer from the Al-Al_2_O_3_ interface^23^. Therefore, the barrier oxide layer at the bottom of the Al_2_O_3_ layer was etched slightly using a mixture of H_3_PO_4_ (15.15 mL) and DI water (500 mL) to open the pores on the back side of the Al_2_O_3_ layer. The yield of the nanoporous Al_2_O_3_ membrane was more than 90%, with uniform hole size and density under optimal process conditions. A 30-nm-thick Au layer was deposited on the open pores of the Al_2_O_3_ layer. The Al_2_O_3_ layer was then removed in a KOH (10 g) and DI water (500 mL) solution to form the Au nanomesh layer. Subsequently, the Au nanomesh was dipped in an aqua regia solution (HCL:HNO_3_:H_2_O = 3:1:2) to smooth the edges of the Au nano-holes. The Au nanomesh layer was then floated on the surface of DI water, and transferred to the MoS_2_/Si substrate by scooping with the substrate. The bulk MoS_2_ layer was etched selectively in a reactive ion etch system with CF_4_ as the carrier gas. Finally, the nanomesh MoS_2_ layer was achieved after removing the Au layer in gold etchant TFA (Transene Company, Inc.).

**Figure 2:**
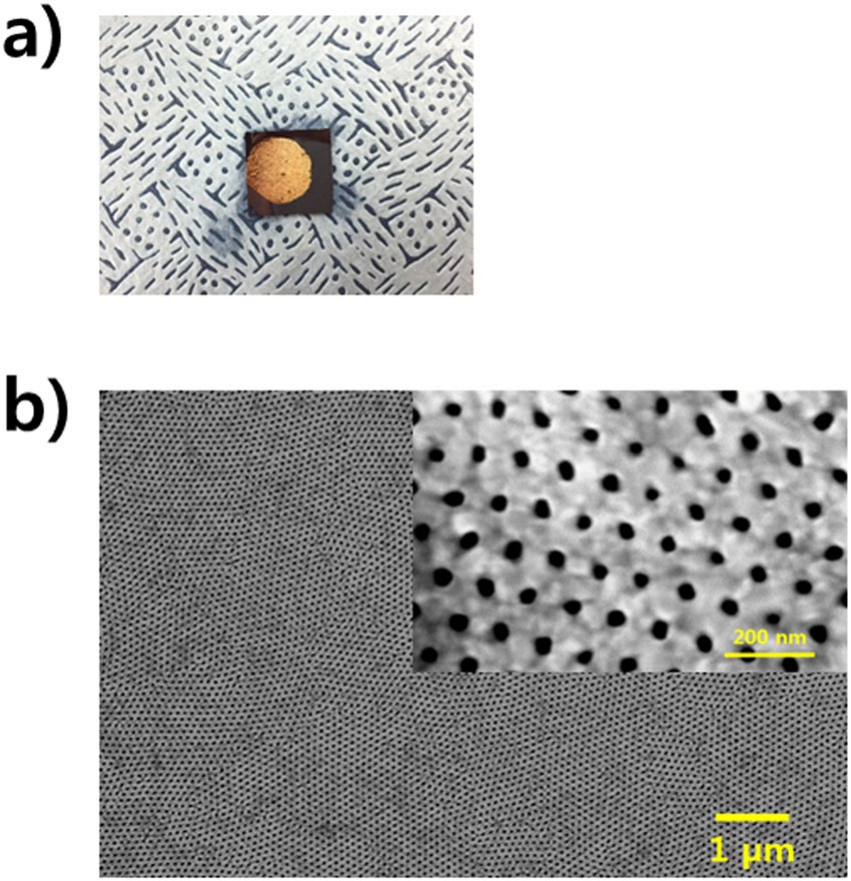
(**a**) Photograph and (**b**) SEM image of the Au nanomesh on a MoS_2_ flake/SiO_2_/Si with a hole size of 30–50 nm and a neck width of 40–50 nm. Inset: Magnified SEM image of the Au nanomesh.

**Figure 3:**
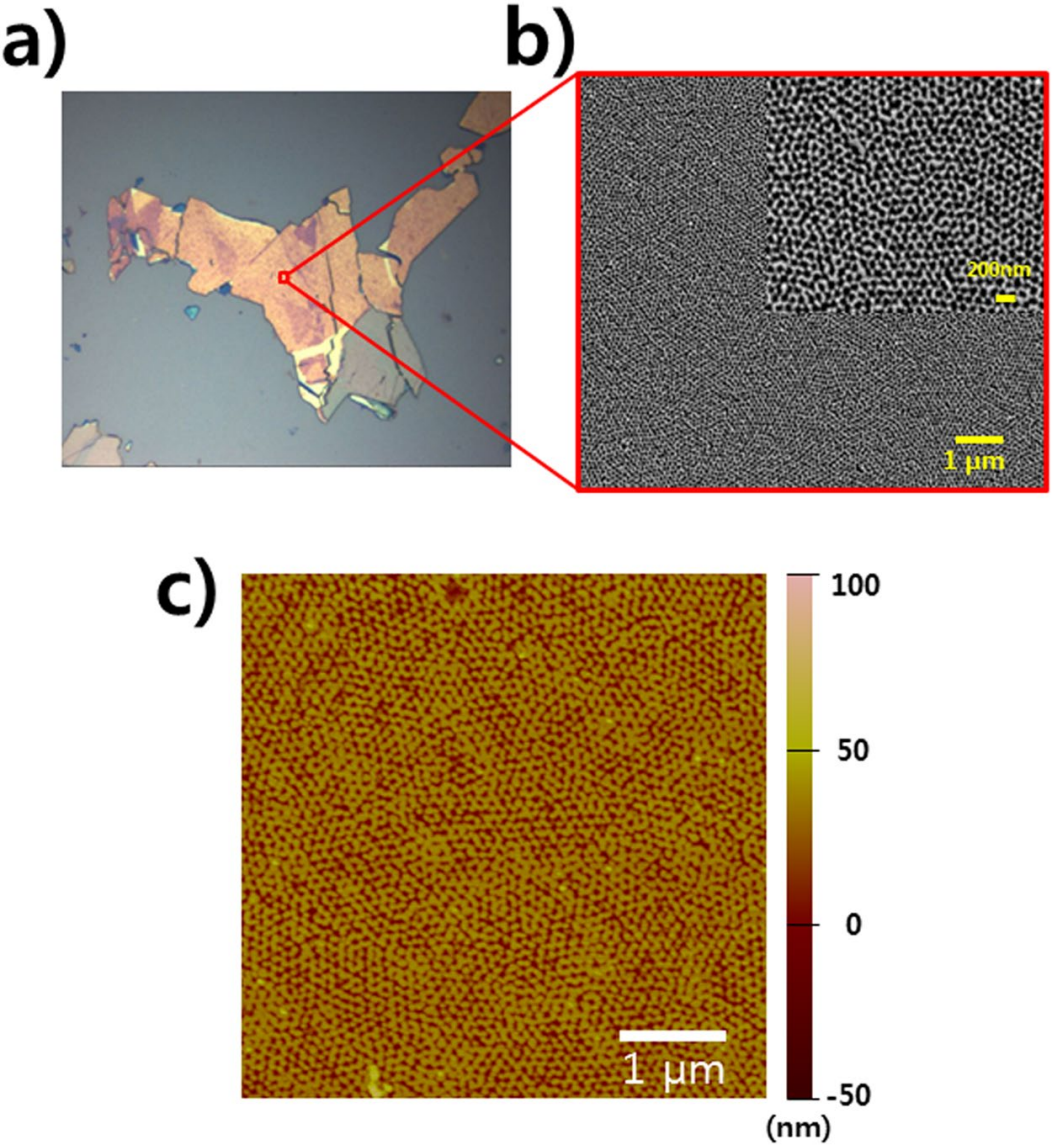
(**a**) Optical microscopy image and (**b**) SEM (**c**) AFM images of a large area. Inset: Magnified SEM image of nanomesh MoS_2_ with a hole size of 80 nm and a neck width of 10 nm after removing Au nanomesh.

The pore size of the nanomesh pattern used in this study was controlled by changing the applied bias when forming the AAO template. The pore diameter and neck width of the AAO template increased from 30 to 200 nm and from 40 to 100 nm with increasing anodizing voltage (Supplementary Fig. S1). The morphology of the nanomesh bulk MoS_2_ materials was examined by scanning electron microscopy (SEM) and atomic force microscopy (AFM). When a 10nm-thick Au template with a hole size of 30 nm and a neck width of 40 nm was used as the masking layer (Fig. 2) and CF_4_ plasma etching was performed for 6 min, nanomesh MoS_2_ with a hole size of 45 nm and a neck width of 30 nm was obtained (Fig. 2a,b). Au was used as a etch mask to fabricate the nanomesh MoS_2_ because it is a highly selective etch mask for oxygen plasma and does not form hybrids by a chemical process^24^. In addition, the Au layer has very high flexibility, so it does not crumble easily under the wet-chemical based transfer process, as shown in Fig. 2. The unsuitable etching process conditions (i.e. excessive plasma power and insufficient etching time) restrict the formation of a perfect nanomesh structure (Supplementary Fig. S2). The AFM image, as shown in Fig. 3c, confirmed that a 32 nm-thick nanomesh MoS_2_ with a mean hole size of 40 nm was determined, which is in good agreement with that obtained from SEM (Fig. 3b).

**Figure 4:**
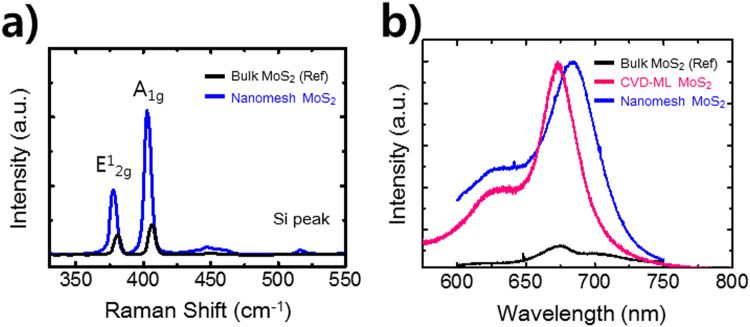
(**a**) Scanning Raman spectrum and (**b**) scanning PL spectrum of bulk MoS_2_, CVD-grown monolayer MoS_2_, and nanomesh MoS_2_.

Raman spectroscopy was performed to examine the structural defects of the nanomesh MoS_2_ with a hole size of 80 nm and a neck width of 10 nm. The bulk MoS_2_ flake possesses the general Raman spectrum with two dominant peaks: the out-of-plane vibration (A_1g_) at 408 cm^−1^ and the in-plane vibration (E^1^_2g_) at 383 cm^−1^ for the bulk MoS_2_ flake. The nanomesh MoS_2_ samples showed a significant red shift for both A_1g_ and E^1^_2g_, corresponding to 5 and 6 cm^−1^, respectively, as shown in Fig. 4a. Moreover, the E^1^_2g_ mode (corresponding to in-plane vibration mode) is preferentially excited for terrace-terminated films, while the A_1g_ mode (corresponding to out-of-plane vibration mode) is dominantly excited for edge-terminated films^25^. The red shift of E^1^_2g_ could be attributed to the abundant terrace-terminated defects in the nanomesh film. The red-shifted out-of-plane (A_1g_) located at 402 cm^−1^ could be explained by the numerous edge-terminated films as well as the doping effects during the process of a MoS_2_ nanomesh^26,27^. For solvents (i.e. gold etchant TFA and aqua regia) used and CF4 treatment using reactive-ion etching (RIE) for the fabrication, no obvious PL and Raman shift was observed (Supplementary Fig. S3), which suggests no doping and no RIE-induced surface defects of the nanomesh MoS_2_. The Raman spectra peak difference values of 25 and 24 cm^−1^ between the two Raman modes observed in the MoS_2_ flake and nanomesh MoS_2_ materials, respectively, were correlated with bulk MoS_2_^28^. The Raman spectra mapping and measured at three different areas showed similar peak positions (Supplementary Fig. S4), indicating the area homogeneity of the optical characteristics of nanomesh MoS_2_.

The optical properties of nanomesh MoS_2_ flakes with a hole size of 80 nm and a neck width of 10 nm were determined by micro-PL spectroscopy. The effect of thickness of bulk MoS_2_ on PL and Raman spectra can be ignored when the thinkness of bulk MoS_2_ is over 6 layers^29,30^. The strain-induced^16,31–33^, structural defects (i.e., grain boundaries, edge, and point defects^21,28,34^) and doping^26^ affect the band structure (i.e., band gap engineering, a direct-to-indirect band gap transition, and semiconductor-to-metal transition) and excitonic optical transition of monolayer and bilayer MoS_2_. Emerging PL was observed in the nanomesh MoS_2_ flake (Fig. 4b), indicating a highly efficient optical transition. Although the bulk MoS_2_ flake as a reference does not exhibit a prominent PL peak, a remarkable PL peak was located at 1.81 eV, which is a slightly lower value than the band gap of monolayer MoS_2_ film at approximately 1.84 eV^10,16,31,32,35^. The broadening of the PL full width half maximum (FWHM) was associated with the radiative transition efficiency. The nanomesh MoS_2_ resulted in a significantly narrower PL FWHM of 59.4 nm as compared to that for the bulk MoS_2_ flake (FWHM of 103.7 nm), although the FWHM of the former is slightly wider than that of monolayer MoS_2_ (FWHM = 36.3 nm)previously obtained by chemical vapor deposition^35^. Although the radiative transition efficiency of nanomesh MoS_2_ is relatively lower than that of monolayer MoS_2_, we believe that this behavior provides evidence of defects-related radiative recombination on the nanomesh structure. The repeatability and reproducibility of AAO method was confirmed by optical characteristics of another nanomesh MoS_2_. (Supplementary Fig. S5) Further simulation and microstructural study, including first-principles calculations and scanning transmission electron microscopy will be necessary to fully understand the effect of structural defects on exciton transition in nanomesh bulk MoS_2_.

## Conclusion

In conclusion, this paper reported a straightforward and practical method for the high yield production of large-area nanomesh bulk MoS_2_ flake using an AAO template. Optical properties of TMDs films can be improved by introducing structural defects in them. Spatial structural defects including edge- and terrace-terminated defects employing a nanomesh structure can be tuned to yield PL enhancement in a bulk MoS_2_ film. Structural defects-induced in bulk MoS_2_ flake using a nanomesh structure effectively lead to a highly efficient radiative transition. These results suggest that a structural defect-induced nanomesh bulk MoS_2_ film could be suitable for the versatile applications such as the photonic, optoelectronics, and photovoltaic applications.

## Method

Preparation of the AAO template: A self-ordered nanomesh Al_2_O_3_ template was generated by the anodization of aluminum. A high purity aluminum disk (Goodfellow, Inc.) of 2 cm diameter was prepared. The aluminum disk was dipped in a mixed solution of perchloric acid and ethanol (1:3) at 1 °C with an applied voltage of 20 V to smoothen the substrate surface. The aluminum disk was then anodized in 0.3 mole of oxalic acid. The anodizing voltage was set in the range of 40 to 120 V to control hole size and neck width. The aluminum substrate was maintained at 1 °C using a cooling stage placed in thermal contact with the substrate. Parameters such as anodizing voltage, anodizing solution, anodizing time and solution temperature affected nanohole size and density of the nanomesh structure. The yield of the nanomesh structure produced by the AAO template was very high for the optimal process conditions. The size and density of the nanohole was uniform on the substrate of 2 cm diameter substrate, except the along the edges.

Nanomesh MoS_2_ fabrication: Bulk MoS_2_ films were exfoliated mechanically from the bulk crystal of MoS_2_ (SPI supplied, purity: >99%) onto SiO_2_/Si with a thickness of 20–200 nm, using scotch tape.

Characterization: The AAO template and nanomesh MoS_2_ flakes were examined by SEM (S-4800, HITACHI). The Raman measurements with the excitation laser line of 488 nm were performed using a Renishaw Raman spectroscope integrated. The power of the excitation laser was maintained at 0.9 mW to avoid heating effects. The Raman emission was collected using a Leica 100 × objective (N.A. = 0.8) and 1800 (for the Raman measurements in Fig. 4a) lines mm^−1^ gratings. The Renishaw Raman spectra with 1200 lines mm^−1^ grating and 1800 lines mm^−1^ grating had a spectral resolution of approximately 1 cm^−1^. A laser with an excitation wavelength of 488 nm and a spot size of 0.75 µm was used. The Si peak was used for normalization. Steady-state PL (LabRam ARAMIS, Horiba Jobin Yvon) measurements were performed at room temperature using a 514 nm-wavelength laser diode with 100 mW power and a beam size of 1 µm. The morphology, hole size, and neck width were evaluated by AFM (VEECO Dimension 3100 + Nanoscope V(Version 7.0), VEECO). For better quality, an image was obtained using a super sharp silicon tip with a radius of curvature of <10 nm (Appnano). The image was taken over a 5 μm^2^ area and a measurement speed of 0.698 Hz.

## Electronic supplementary material

Supplementary Information
