# Supplementary material for: Structural defects in a nanomesh of bulk MoS2 using an anodic aluminum oxide template for photoluminescence efficiency enhancement
Source: Sci Rep. 2018 Apr 27;8:6648. doi: 10.1038/s41598-018-25045-z (PMC5923261; doi:10.1038/s41598-018-25045-z)

Structural defects in a nanomesh of bulk MoS_2_ using an anodic aluminum oxide template for photoluminescence efficiency enhancement

TaeWan Kim^1,†^, DongHwan Kim^1,2,†^, Chan Ho Choi^2,3^, DaeHwa Joung^1,4^, JongHoo Park^4^, Jae Cheol Shin^2,*^, and Sang-Woo Kang^1,5,*^

^1^Advanced Instrumentation Institute, Korea Research Institute of Standards and Science, Daejeon 34113, South Korea

^2^Department of Physics, Yeungnam University, Gyeongsan 38541, South Korea

^3^National Institute for Nanomaterials Technology, Pohang 37673, South Korea

^4^Department of Electrical Engineering, Kyungpook National University, Daegu 41566, South Korea

^5^Department of Next-generation Device Engineering, University of Science and Technology, Daejeon 34602, South Korea

^†^ These authors contributed equally to this work

^*^ Correspondence and requests for materials should be addressed to J.C.S. (email: [jcshin@yu.ac.kr](mailto:jcshin@yu.ac.kr)) and S.K. (email: [swkang@kriss.re.kr](mailto:swkang@kriss.re.kr))

Keywords: nanomesh MoS_2_, Structural defects, anodic aluminum oxide, photoluminescence

**Supplementary Figure S1. SEM images of the AAO templates with a) hole size of 30 – 50 nm and neck width of 40 – 50 nm with anodizing voltage of 40V and b) hole size of 180 – 200 nm and neck width of 90 – 100 nm with anodizing voltage of 150V.**


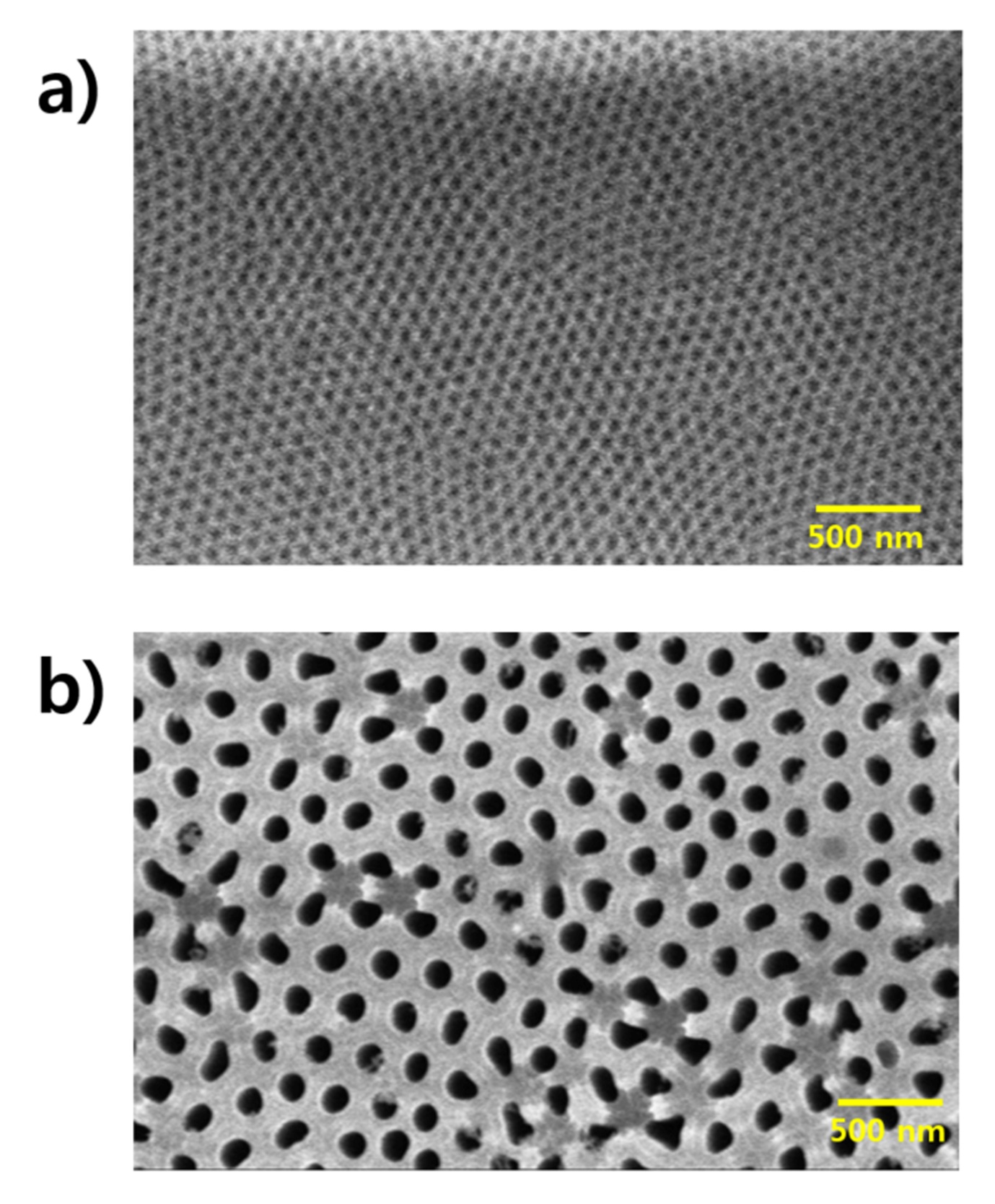


**Supplementary Figure S2. SEM images of nanomesh MoS_2_ with the unsuitable etching process conditions.**


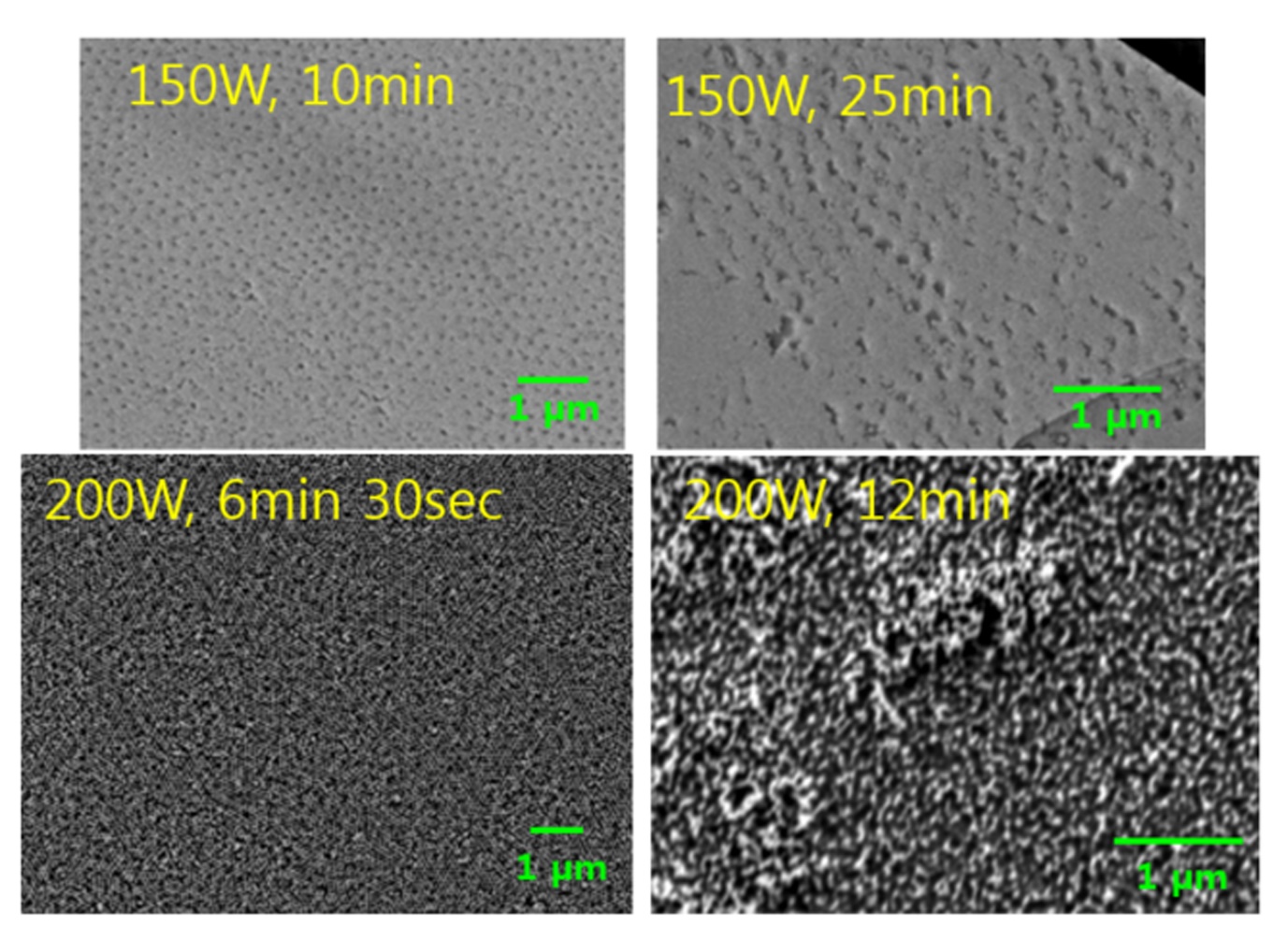


**Supplementary Figure S3. Raman spectra and photoluminescence (at room temperature) results of bulk MoS_2_ after (a) immersing in gold etchant TFA and aqua regia for 10 second (b) CF_4_ treatment using RIE**

**(a) Doping effect after dipping gold etchant and Aqua regia treatment**

**
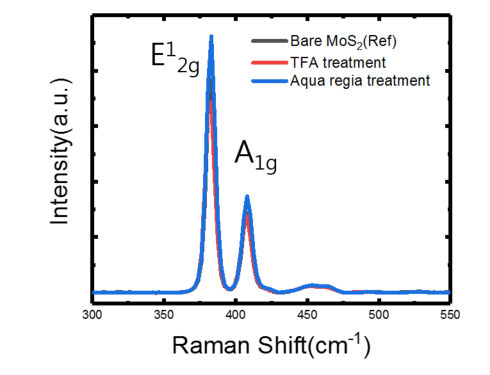

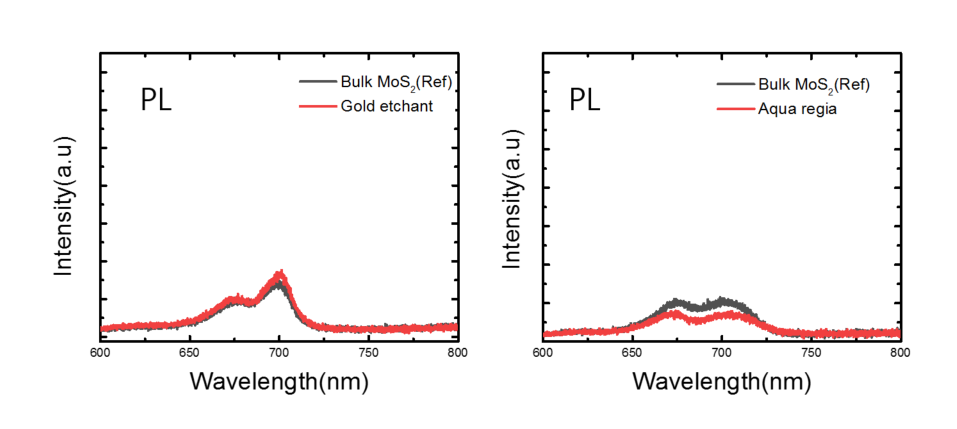
**

**(b) Surface defect effect after CF4 treatment**

**
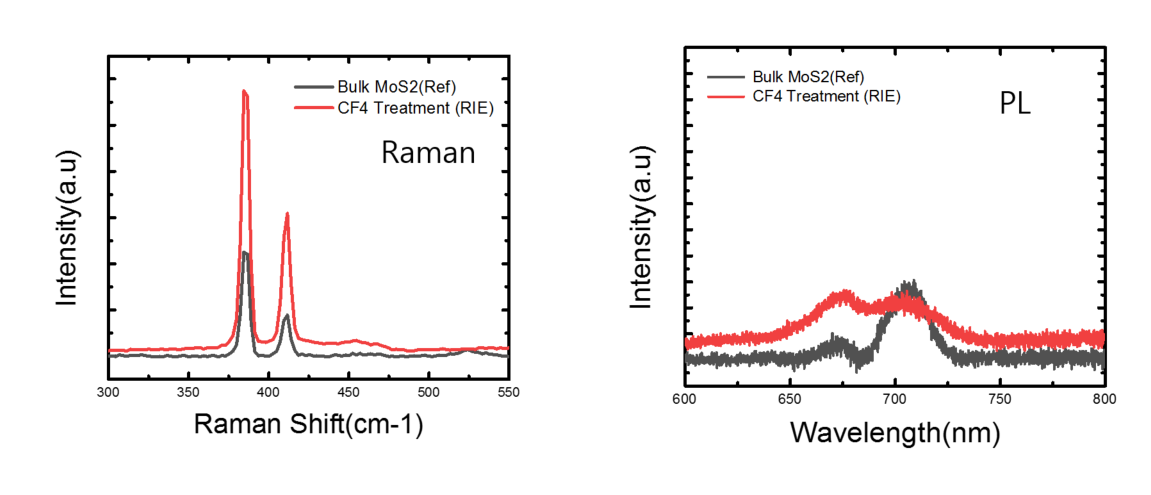
**

**Supplementary Figure S4. (a) Raman spectra results of nanomesh MoS_2_ at the various different areas. (b) Raman mapping result**

**(a)**


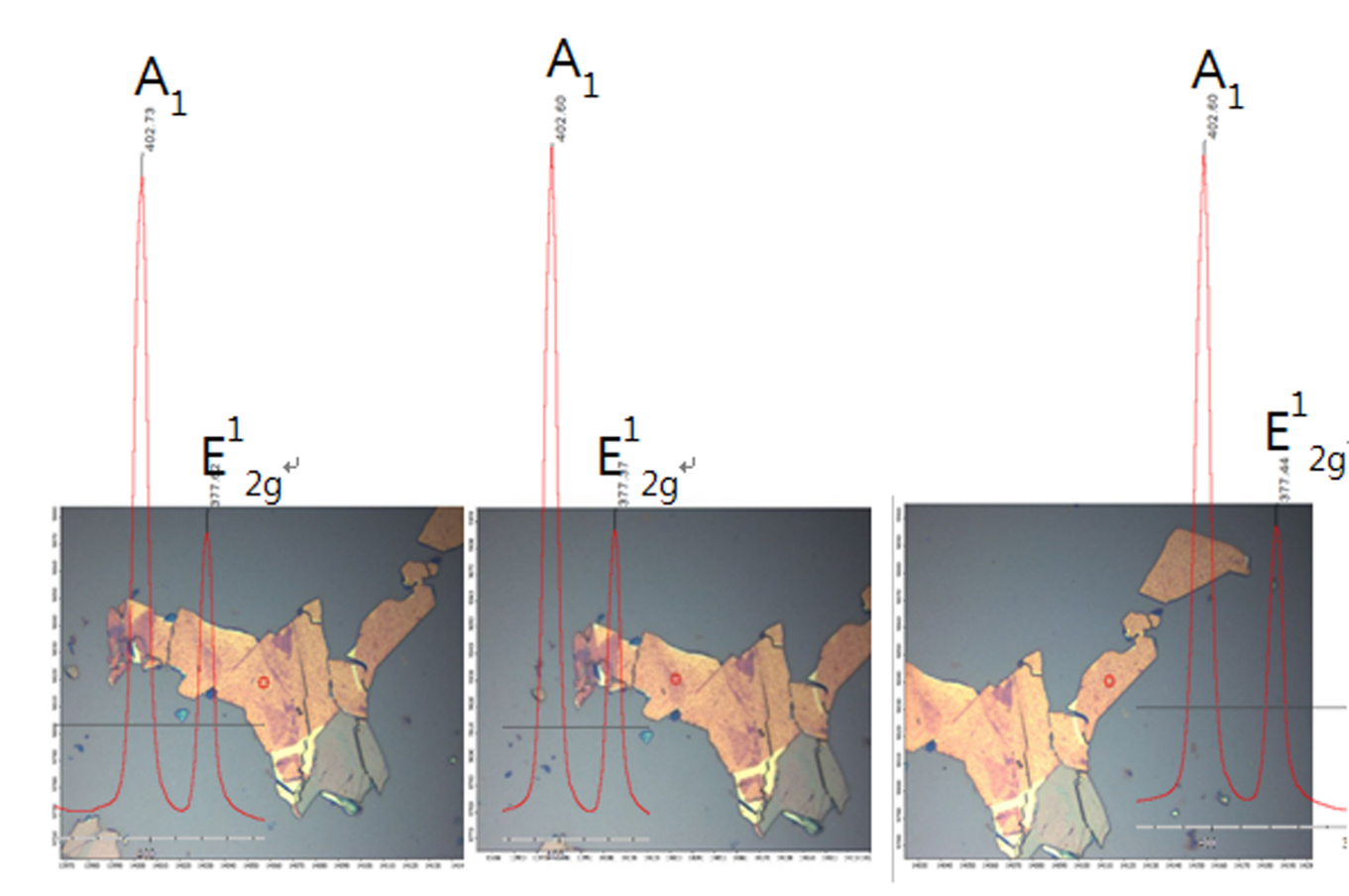


**(b)**


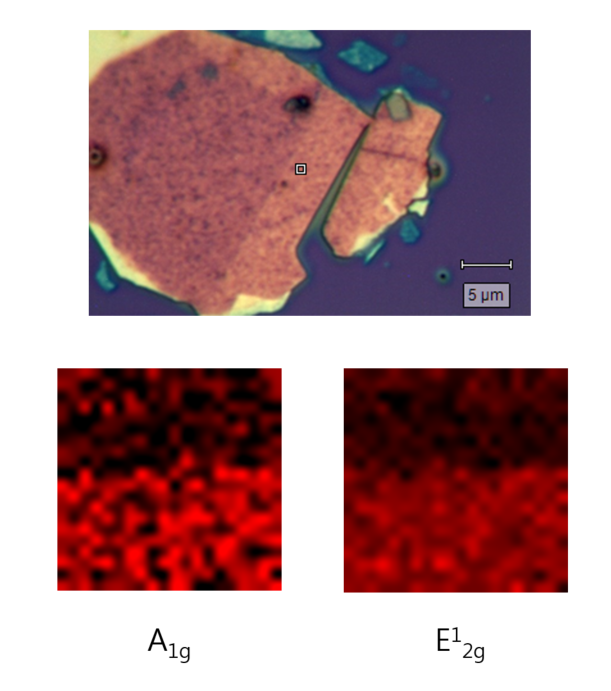


**Supplementary Figure S5. PL and Raman spectra results of nanomesh MoS_2_ at different areas for the repeatability of AAO method.**


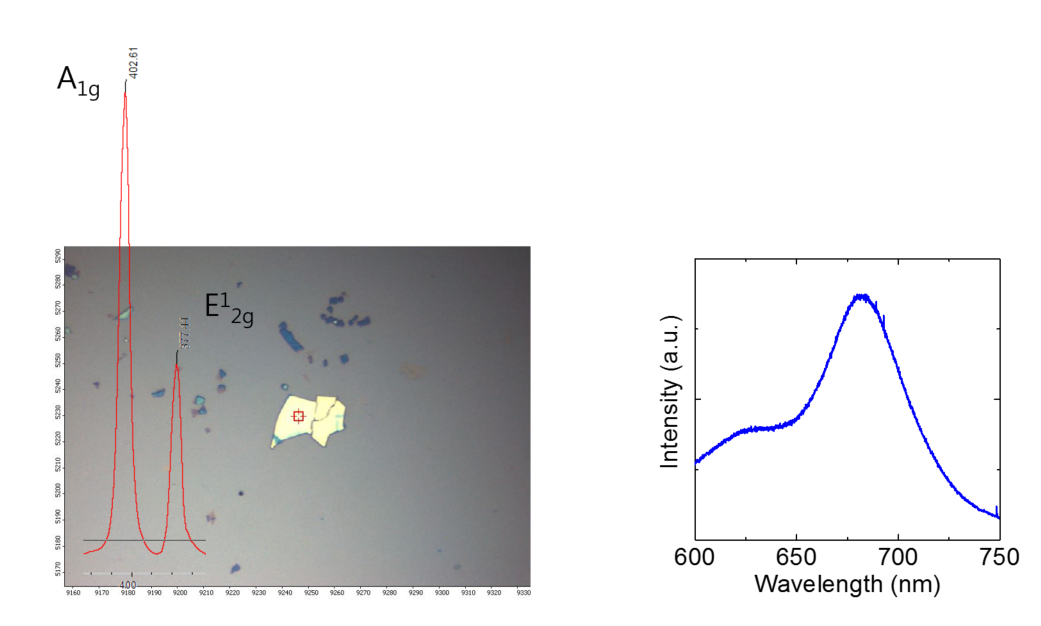

Supplement: Supplementary file 1 — Supplementary Information [file 41598_2018_25045_MOESM1_ESM.doc]
